# Dynamic Manipulation of Chiral Domain Wall Spacing for Advanced Spintronic Memory and Logic Devices

**DOI:** 10.1021/acsnano.4c02024

**Published:** 2024-05-17

**Authors:** Jae-Chun Jeon, Andrea Migliorini, Lukas Fischer, Jiho Yoon, Stuart S. P. Parkin

**Affiliations:** † Max Planck Institute for Microstructure Physics, D-06120 Halle (Saale), Germany; ‡ Martin Luther University Halle-Wittenberg, 06108 Halle (Saale), Germany

**Keywords:** racetrack, spintronics, domain wall motion, geometrical effects, memory, logic

## Abstract

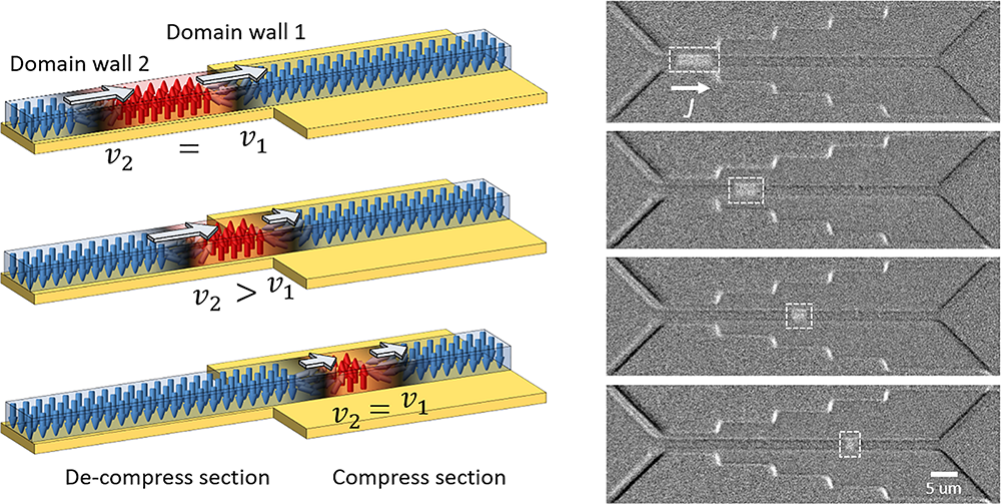

Nanoscopic magnetic domain walls (DWs), via their absence or presence, enable highly interesting binary data bits. The current-controlled, high-speed, synchronous motion of sequences of chiral DWs in magnetic nanoconduits induced by current pulses makes possible high-performance spintronic memory and logic devices. The closer the spacing between neighboring DWs in an individual conduit or nanowire, the higher the data density of the device, but at the same time, the more difficult it is to read the bits. Here, we show how the DW spacing can be dynamically varied to facilitate reading for otherwise closely packed bits. In the first method, the current density is increased in portions of the conduit that, thereby, locally speeds up the DWs, decompressing them and making them easier to read. In the second method, a localized bias current is used to compress and decompress the DW spacing. Both of these methods are demonstrated experimentally and validated by micromagnetic simulations. DW compression and decompression rates as high as 88% are shown. These methods can increase the density with which DWs can be packed in future DW-based spintronic devices by more than an order of magnitude.

## Introduction

The latest developments in domain wall (DW)-based devices^1−10^ have attracted much attention for their potential for advanced memory as well as logic applications. Many of these innately spintronic devices rely on the current-induced motion of domain walls (CIDWMs), the data bits, along magnetic nanowire conduits, often referred to as “racetracks”. The latest findings have shown that spin-orbit torques (SOTs), which are derived from spin currents generated by the conversion of charge currents injected into a spin Hall layer adjacent to perpendicularly magnetized racetracks, can be highly efficient in moving the DWs in these racetracks when these DWs are chiral with a Néel structure. The velocity of the DWs, *v*_CIDWM_, increases with the applied current density when this current density exceeds a threshold value, i.e., *J* > *J*_th_, and can attain speeds exceeding 1 km/s in synthetic antiferromagnet (SAF) racetracks.^11−14^ These very high speeds, thereby, allow for very fast operation. Together with the innate nonvolatility of the magnetic structures, DW logic and memory technologies have great potential for going beyond today’s complementary metal-oxide-semiconductor (CMOS) technologies.^15−19^

DW-based devices are formed from magnetic nanowires that have three main components: (i) writing ports, (ii) a bit storing body (Cache), and (iii) reading ports.^15^ There has been extensive research concerning the writing and reading of DWs. The writing process includes the creation and motion of DWs.^20−24^ The reading of DWs via magnetic tunnel junctions (MTJs) built into or adjacent to the racetracks is a preferred method due to the substantial signals that thereby arise.^15,17,21−24^ However, a major challenge in DW-based devices is to achieve the closest possible packing of the DWs so as to attain high densities and, at the same time, to be able to read the DWs when they are densely packed together. It is difficult to build MTJs with diameters as small as the smallest possible width of such DWs (a few nanometers). Here, we demonstrate how to dynamically increase the spacing between the DWs to allow them to be read more reliably. We show that the spacing between DWs can be compressed by more than 80% for storing data bits and then decompressed for easier reading. We present two methods for controlling the DW spacing. First, passive manipulation, where the geometry of the racetrack device is engineered so as to locally change the current density and, thereby, allow for the acceleration or deceleration of a given DW or DWs. We demonstrate the compression and subsequent decompression of the DW spacing in a SAF racetrack by almost an order of magnitude. Second, active manipulation where the local current density is varied by applying a local bias current. These schemes are demonstrated both experimentally and via micromagnetic simulations. Using these schemes, we propose advanced DW-based devices with a higher memory capacity and precision bit reading.

## Results and Discussion

In a racetrack device with multiple number of DWs, it has been shown that the DWs can be moved synchronously at the same speed upon the injection of a current pulse assuming a uniform racetrack and that the DWs have a chiral structure for motion via SOT.^14,21^ This implies that the operation energy per bit can be significantly lower than that of standard magnetic random access memories. However, because of the synchronous motion of multiple DWs, manipulating the spacing between DWs is not trivial. Our approach is to make the motion of a given DW locally faster or slower than the another DW so that the spacing between them can be compressed or decompressed.

**Figure 1:**
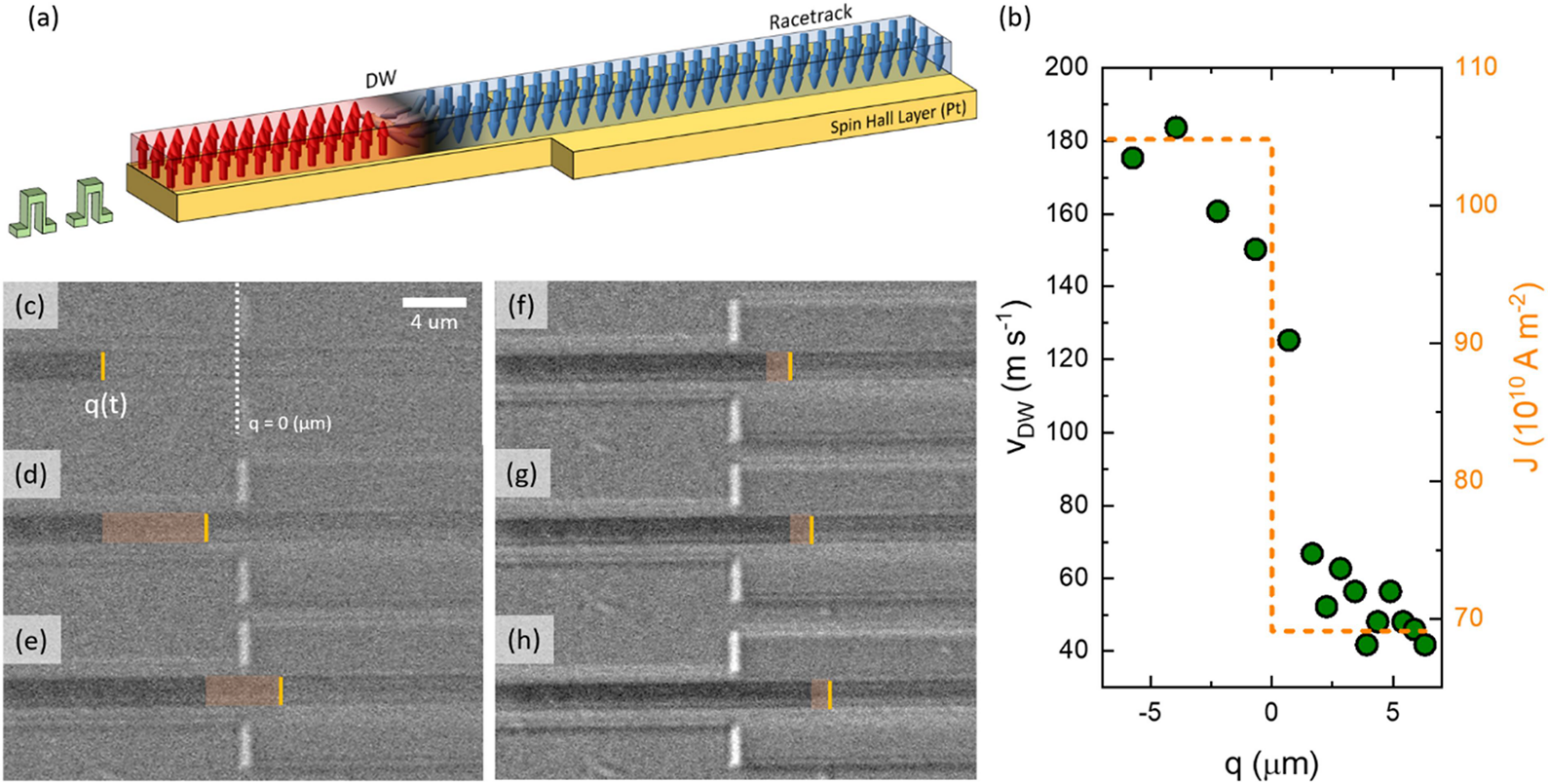
(a) Schematic illustration of a passive DW controller. The device is divided into two regions so that the width of the spin Hall layer is wider in one section compared to the other, but the magnetic nanowire is uniform in width. (b) Velocity of the DW measured at various points along the nanowire. For *q* > 0 μm, at 11 V pulse (5 ns pulse), the current density drops (105 MA cm^–2^ to 69 MA cm^–2^) due to the increased width of the Pt layer. The drop in the current density lowers *v*_CIDWM_ from ∼180 m s^–1^ to ∼50 m s^–1^. (c–h) Differential Kerr microscopy images after (c–e) injecting five consecutive pulses and (f–h) injecting 10 consecutive pulses (10 ns long, 10 V). The orange shades indicate the distance of the DW shift. Note that the speed of the DW was 88 and 36 m s^–1^ in section 1 (narrow region) and section 2 (wide region), respectively.

An elegant approach for the local passive control of *v*_CIDWM_ is to modify the driving current density locally so that a given DW travels faster or slower. This can be affected in various ways, e.g., by locally changing the detailed structure of the racetrack, such as by adding one or more metallic layers, but perhaps the simplest approach is to vary the width of the racetrack so that the current density is locally changed. However, when there are noncontinuous features along the magnetic nanowire, these may create significant pinning potentials that make the CIDWM difficult and noncontrollable. In order to mitigate this issue, we prepared conduits using a two-step etching process. Here, we mostly use conduits formed from a conventional SAF structure (30Pt–3Co–7Ni–1.5Co–8Ru–3Co–7Ni–3Co; units in Å) as it offers excellent stability and speed.^14^ In a first step, we define the magnetic nanowire by using an etching process that etches the heterostructured magnetic layers (Co–Ni–Co–Ru–Co–Ni–Co) down to the lower spin Hall layer (Pt). For such precise control, the in situ detection of the etched material is performed during the etching process using secondary ion mass spectroscopy (SIMS). Then, in a second step, the spin Hall layer is etched to have wider local sections, thereby locally reducing the current density. Using this approach, one can passively control the net cross-sectional area of the device while mitigating any damage to the edges of the magnetic layer. See Methods and SI (Figures S1 and S2) for further details and Figure 1a for a schematic illustration of the device. In such a structure, for an injected current pulse with a given amplitude, the current density will be lower in the wider region so that *v*_CIDWM_ will be correspondingly slower in this region since the speed of the DW is proportional to the current density (*v*_CIDWM_ ∝ *J*). The variation of *v*_CIDWM_ along the magnetic wire is shown in Figure 1b. *v*_CIDWM_ clearly drops from ∼180 m s^–1^ in section 1 (*J* = 106 MA cm^–2^) to ∼50 m s^–1^ in section 2 (*J* = 70 MA cm^–2^). Note that the speed of the DW motion is calculated by dividing the distance traveled (evolution of Kerr contrast after the injection of a series of current pulses) by the total applied pulse length.

The motion of an injected DW along the nanowire is visualized by differential Kerr microscopy with consecutive pulses, as shown in Figure 1c–h. The images clearly show both the patterned Pt layer with two different widths and the magnetic nanowire, which is uniform in width. Moreover, magnetic contrast (dark gray) is only observed in the magnetic nanowire region and not from the Pt layer, indicating a successful etching process. *v*_CIDWM_ vs *J* in each section of the device is shown in Figure S3. Yellow lines mark the position of the DW after injecting the pulses (orange shading indicates the travel distance). In section 2 (Figure 1f–h), the number of injected pulses was doubled (10 pulses, 10 ns long, 10 V) to acquire measurable optical contrast from the DW propagation. It is clear that the shift range (orange shading) of the DW at a given pulse amplitude is noticeably smaller in section 2. The speed of the DW was 88 and 36 m s^–1^ in section 1 (narrow region) and section 2 (wide region), respectively. It is noteworthy that the DW does not experience any pinning when the DW shifts into the region where the local current density varies, owing to the geometry of the device—a straight magnetic wire with an extended spin Hall layer for local current density control.

**Figure 2:**
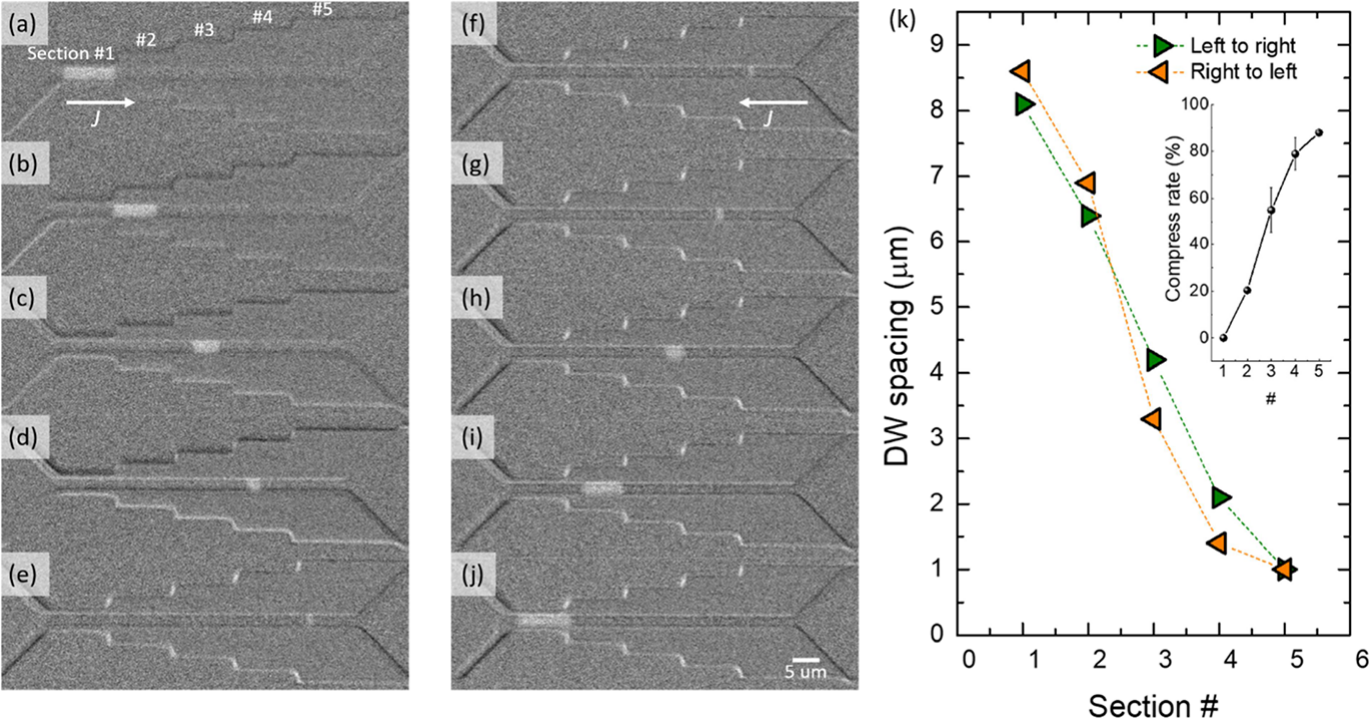
Sequential snap shots of current-induced DW motion in multisectioned device. (a)–(e) Forward propagation of two DWs. Series of 22 V, 5 ns long pulses are injected to position the DWs in each section (section 1, left-narrow to section 5, right-wide). The DW spacing in each section were 8.1, 6.4, 4.2, 2.1, and 1 μm, respectively. (f)–(j) Backward propagation of two DWs. The shift-back process was achieved by 5 ns long pulses with an amplitude of negative 22 V. The DW spacing was decompressed from 1 μm ± 0.5 to 8.6 μm ± 0.5 μm upon the return of the DWs to the original position. The DW spacings in each section were 1, 1.4, 3.3, 6.9, and 8.6 μm, respectively. (k) Summary of the DW spacing and compression rate changes in different sections (green: motion from left (high *J*) to right (low *J*); orange: motion from right (low *J*) to left (high *J*)).

Now we demonstrate manipulation of the DW spacing in a SAF racetrack. A device is prepared in which the spin hall layer has five sections whose widths are progressively increased in steps of 4 μm from left to right (from 4 to 20 μm), while the width of the uniform magnetic conduit is 2 μm. The operation of the device is illustrated in Figure 2a–j. First, two DWs, separated by 8.1 ± 0.5 μm, are injected. Then, a series of nanosecond-long current pulses was applied to shift the DWs from left to right. The spacing between the DWs is successively decreased as they enter each successive section due to the increased width of the Pt layer, which reduces the current density in this layer. Indeed, the spacing is reduced to only 1 μm ± 0.5 μm in the last section (see Figure 2a–e). The compression rate, defined by 100 × (*l*_ini_ – *l*_comp_)/*l*_ini_, where *l*_ini_ and *l*_comp_ are the DW spacing at initial and compressed states, is ∼88%. Importantly, the DW spacing returns to its original size when the DWs are shifted back to their original position (Figure 2f–j). Note that when the multistep device is made by patterning both the magnetic and Pt layers in a single etch process, each step creates strong pinning of the DW due to the sudden change in the DW’s length, which hampers the continuous CIDWM (see Figure S4).

**Figure 3:**
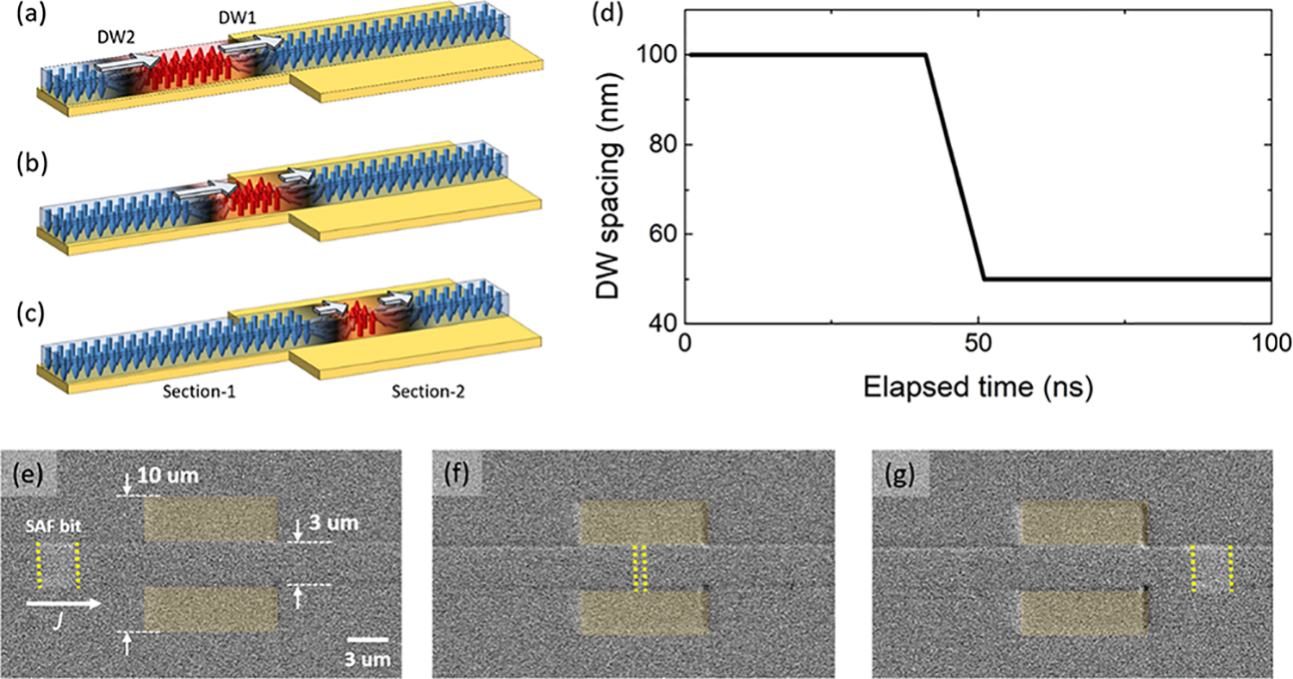
(a–c) Model for a passive DW spacing controller. The illustrations depict the shrinking of the DW spacing as it is moved from a narrow section 1 to a wider section 2 of the racetrack. As DW1 enters section 2, where the current density is lower, *v*_DW1_ (speed of DW1 at given current pulse) slows down, while DW2 keeps its original speed. The length of the white arrows in the illustrations represents the speed. As a consequence, the DW spacing shrinks. (d) Numerically simulated result for the case when a 100 nm wide bit enters a region with half the current density, i.e., half *v*_CIDWM_. (e–g) Demonstration of DW spacing control in a decompression–compression–decompression type device. The Pt width is enlarged in the middle of the structure (racetrack width of 3 μm; Pt width of 10 μm).

In order to numerically estimate the DW spacing when a DW enters the compression region, we use a simplified model in which the DW speed varies linearly with *J*. Phenomenologically, the slope of *J* vs *v*_CIDWM_ is nearly linear for intermediate *J* (e.g., 60 MA cm^–2^ < *J* < 125 MA cm^–2^ in Figure S3) but nonlinear near the threshold *J* for motion and at high *J*. Consider two DWs in the first section. When the first DW, i.e., DW1, enters section 2, its speed slows down due to the drop in the current density while the second DW (DW2) maintains its speed. This compresses the spacing until DW2 enters the same region (Figure 3a–c). On the contrary, the spacing is decompressed when the DWs are moved back. In reality, the nonlinearity in the relationship between *J* and *v*_CIDWM_ caused by the ambient environment (e.g., temperature, magnetic field, etc.), Joule heating, line edge roughness, and any other defects will affect the quality of the control and can even lead to the collapse of the adjacent DWs. Figure 3d shows a numerically simulated result. For the numerical simulation, we have set *v*_CIDWM_ = 10 m s^–1^ in the narrower (decompress) region while *v*_CIDWM_ = 5 m s^–1^ in the wider (compress) region for simplicity. In this case, the DW spacing compresses by a factor of 2, i.e., the 100 nm initial DW spacing compresses to 50 nm in the controlled region. Table S1 in the Supporting Information shows results from three experimental operations. The mean value of the difference between the estimate and the experiment was 18 ± 5% (see Figure S8 for further discussion).

We performed passive DW spacing control experiments with decompression–compression–decompression type devices as well. For these experiments, we designed devices with different racetrack width to Pt width ratios (1:2, 1:3.3, and 1:4—see Figure S5). The compression rates in each device were found to be 59, 72, and 78%, respectively (Figure S6). Figure 3e–g shows the process of decompression–compression–decompression in a device with a 1:3.3 ratio (3 μm wide racetrack: 10 μm with Pt). Note that the exposed Pt area is shaded in yellow. It clearly shows the DW spacing (DWs marked with green dot-lines; ∼3 μm spacing) compresses (nearly invisible to the microscope; ∼0.84 μm spacing) and decompresses as it travels through the structure. Furthermore, we shifted a relatively small DW spacing (2.2 μm) into the compressed region in the device with a width ratio of 1:4 (Figure S7). In this case, the DW spacing was expected to be 480 nm in the compressed region according to the compression ratio of the device. When both DWs enter the compressed region, the optical contrast of the DW spacing becomes indistinguishable from the background. We then observed the recovery of the DW spacing in the decompress region by injecting current pulses to push the DWs outside the compress region. This experiment confirms the stability of the DW spacing even at the nanometer scale in a SAF racetrack, which, to our knowledge, has never been demonstrated before.

**Figure 4:**
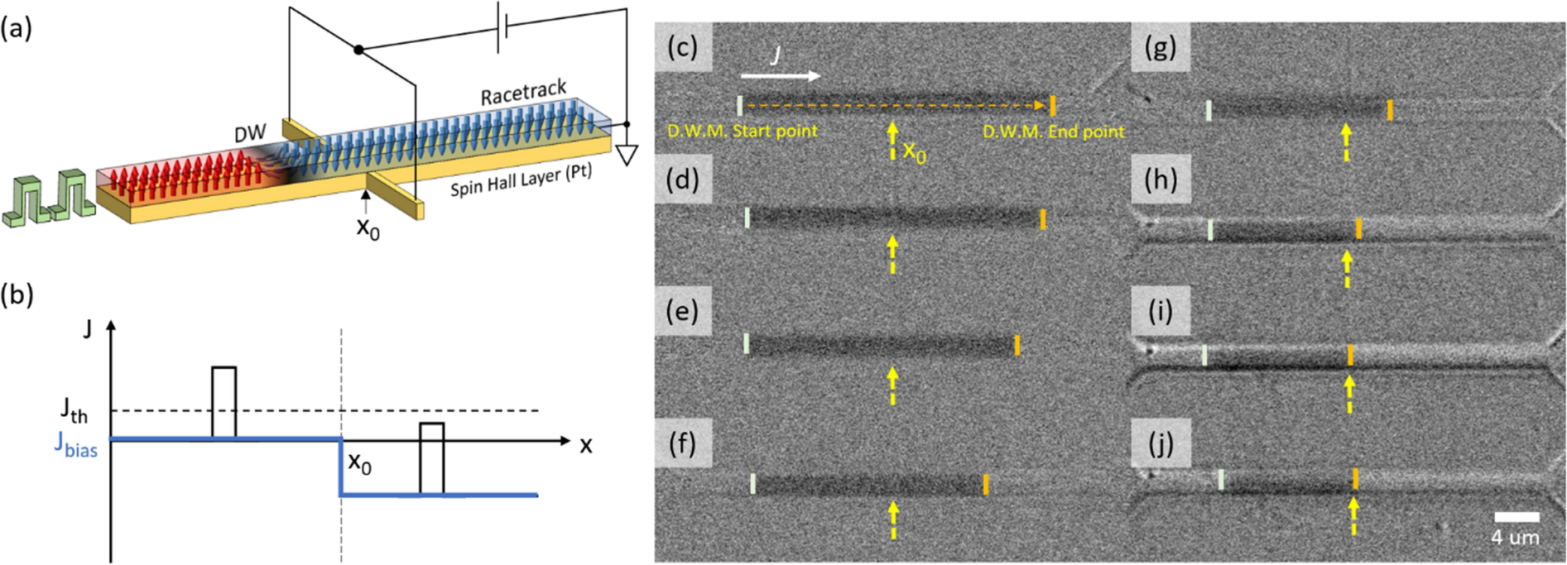
(a) Schematic illustration of active DW spacing control device. The bar across the racetrack is used for the bias point. This, in principle, can be placed under or over the racetrack. (b) Electrical potential along the racetrack upon application of *V*_bias_. *x*_0_ indicates the bias point. Upon reverse bias, the applied pulse amplitude (forward motion) is lower than the threshold level. This stops the motion of the DW into the bias region. (c) DWM with 8 V; 5 ns; 100 pulses at zero bias. White and orange bars indicate the starting and end points of the DW motion. After placing the DW to the original position, the same pulses were injected upon application of bias (d) −1, (e) −2, (f) −3, (g) −4, (h) −5, and (i) −6 V. At zero bias, the DW was shifted 29 μm upon 100 injected pulses, so that *v*_CIDWM_ = 58 m s^–1^. As the reverse bias is increased, the *v*_CIDWM_ in the biased region was found to be 58 m s^–1^, 50 m s^–1^, 42 m s^–1^, 10 m s^–1^, and 0 m s^–1^ with *V*_bias_ = −1, −2, −3, −4, −5, and −6 V, respectively. (j) Note that even after injecting 1000 pulses, the DW stays at the bias point. This confirms that the DW does not propagate into the bias region at *V*_bias_ = −6 V.

In the following, we present a method to actively manipulate the spacing between the DWs. For this, we have constructed a narrow bar from the spin Hall layer to apply a local bias current. Figure 4a,b shows schematic illustrations of the device and the working principle, respectively. By adjusting the level of the negative bias, the local current density in the bias region can be varied. Note that the negative bias current can cause the DW to move against the direction of the positive current pulse and can be used to position the DW at a desired position. An example is given in Figure 4c–i. Here, a DW initially positioned at the left end of the device is moved with current pulses to the right. At the same time, a local bias voltage is used to slow down the DW. Note that at *V*_bias_ = −6 V, the DW moves against the direction of the shift pulse until it is blocked at the leftmost edge of the bias region. The complete stopping of the DW was confirmed by injecting 1,000 pulses with *V*_pulse_ = 8 V under *V*_bias_= −6 V, as shown in Figure 4j. Such active pinning can be used to decrease the spacing between DWs. For example, if the first DW is stopped by *V*_bias_ and the second DW keeps its original speed, then the spacing will be decreased. Depending on the length and amplitude of the injected current pulse, the final spacing between the DWs can be precisely manipulated. Compression of the DW spacing of up to 83% was achieved. An example of the active manipulation of DW spacing is shown in Figure S9.

**Figure 5:**
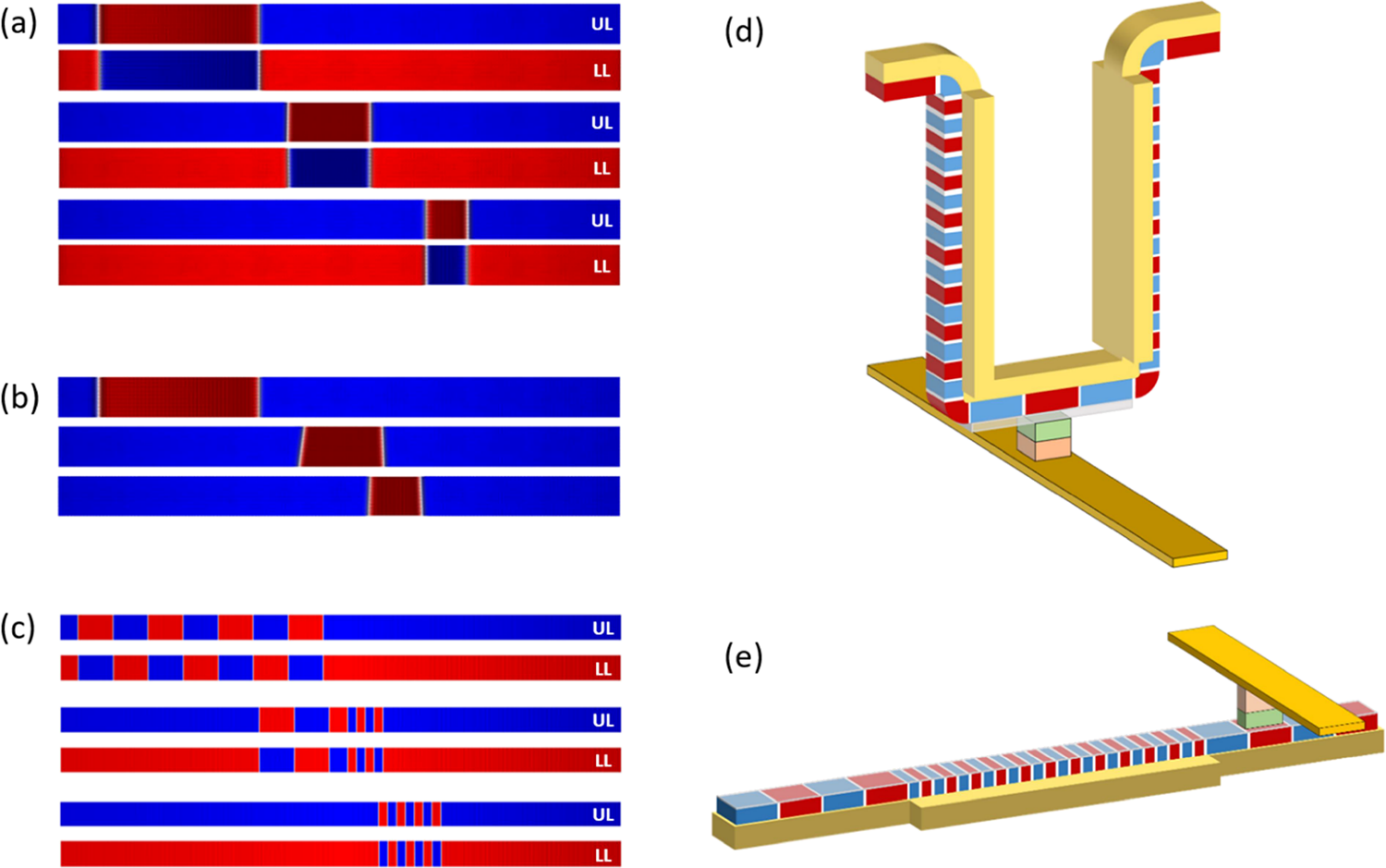
Micromagnetic simulations for DW spacing control in (a) SAF and (b) FM racetracks. UL and LL denote upper and lower layers, respectively. The size of the racetrack was set to be 700 nm (length) by 50 nm (width). In the SAF, the 200 nm DW spacing shrinks to 53 nm in section 2, where the current density drops by a quarter. On the other hand, in the FM case, the 200 nm DW spacing shrinks to ∼70 nm in section 2. Note that the minimum DW spacing is larger for a FM due to the existence of the stray field. (c) Demonstration of the compression of multi-DWs in the SAF racetrack. High bit density racetrack memory device with (d) 3D and (e) linear-type structures. The extended spin Hall layer acts as a bit compressor, which can maximize the density of DW bits.

As Kerr microscopy technology lacks high spatial resolution, we further confirm the validity of our work in nanometer-scale devices via micromagnetic simulations (mumax3 platform) for both ferromagnetic (FM) and SAF racetracks. For the simulations, a racetrack with dimensions of 700 nm by 50 nm by 1 nm (3 nm for SAF) was used. The following parameters were used: *M*_s_, *K*_u_, *A*_ex_, *D*_ind_ = 0.6 MA m^–1^, 1 MJ m^–3^, 1 × 10^–11^ J m^–1^, 1 mJ m^–2^, respectively. Current density was set to be different in the 2 regions (20 MA/cm^2^ for *x* < 0 and 5 MA cm^–2^ for *x* > 0). Figure 5a shows snapshots of the simulation results for the SAF racetrack. UL and LL indicate the upper and lower layers of the SAF, respectively. Initially, a 200-nm-wide DW spacing was set (20 MA cm^–2^). Then, as it propagates into the region with the lower current density (5 MA cm^–2^), the DW spacing is compressed. When both DWs entered the low current density region, the final size of the DW spacing was 52 nm. Figure 5b shows the case for the FM racetrack. In this case, although the DW spacing was compressed to ∼50 nm momentarily, the final size of the DW spacing was observed to be ∼70 nm due to dipolar field effects. See the attached Videos S1 and S2 for the DW spacing compression in FM and SAF racetracks. Furthermore, we simulate the compression of the multi-DWs in an SAF racetrack. Figure 5c shows snapshots of the simulation results for the compression of 8 DWs. The initial spacing between the DWs was set to be 200 nm for all 8 DWs. As the DWs enter the low current density region, the spacing between the DWs is found to be 52 nm, as shown in Figure 5a.

From the experimental and simulation results shown in this work, we propose advanced designs of racetrack memory that can store a higher data bit density, i.e., closely packed DWs. In vertical- and linear-type SOT-based racetrack memory, a widened spin Hall layer, which serves as a DW spacing compressor, can be added to the bit-storing body. On the other hand, a narrower spin Hall layer serves as a DW spacing decompressor. When DWs with small spacing shift into the decompress region, the spacing between adjacent DWs is expanded. This will drastically lower the error in bit-reading by a read sensor (e.g., MTJ). As an example, if *v*_CIDWM_ = 100 m s^–1^, it leaves only a 500 ps read margin to sense the signal from shifting to a 50-nm DW spacing. However, if the DW spacing is decompressed to 200 nm, there will be a 2 ns read margin, which will dramatically reduce the read error rates. Figure 5d,e illustrates designs for proposed DW-based devices with 3D and linear geometries, respectively.

## Conclusions

In summary, we have presented passive and active manipulation of the spacing between chiral DWs in SAF magnetic nanowires with PMA. For passive manipulation, we geometrically engineered the width of the spin Hall layer (Pt) while keeping the magnetic layer intact. By locally varying the width of the Pt layer, we were able to control the local current density. We demonstrated the compression and decompression of DW spacing with Pt-wing-stepped devices. We successfully showed a DW spacing compression ratio of 88%. Importantly, we compressed a DW spacing beyond the Kerr detection range and decompressed it to its original size, which confirms the stability of nanometer scale DW spacing, i.e., high bit density, in the SAF racetrack. Additionally, we actively controlled the local current density by a locally applied bias. We experimentally confirmed that the active DW spacing compression rate can be as high as 83%. Investigations using micromagnetic simulations validated that the spacing between chiral DWs can be controlled on the sub-100 nm scale by the manipulation of the local current density. Our work allows for advanced DW-based device technologies with higher bit densities, which will further increase both the scientific and technological interest in DW-based technologies for memory and in-memory computing.

## Methods

### Sample Preparation and Device Fabrication

The films were prepared in a specially designed physical vapor deposition system MANGO (multisource, atomically engineered, next generation, alloys, and compounds deposition system). The system has a base pressure of <10^–9^ Torr. The deposition was carried out at room temperature using magnetron sputtering at an Ar pressure of 3 mTorr. The film structure was as follows: (Substrate) Si-SiO_2_ || 20 TaN | 30 Pt | 3 Co | 7 Ni | 3 Co | 30 TaN for FM and (Substrate) Si-SiO_2_ || 20 TaN | 30 Pt | 3 Co | 7 Ni | 1.5 Co | 8 Ru | 3 Co | 7 Ni | 3 Co | 30TaN (units in A) for SAF. The as-deposited films were patterned using e-beam and photolithography and etched using argon ion beam etching processes. The e-beam lithography (JEOL EBL (100 kV)) was carried out with an e-beam resist (ARN-7520-18). The etching was performed at a beam incidence angle of 30 deg with respect to the normal to the film’s plane to minimize the redeposition of etched materials while maximizing the etching uniformity (scia coat 200 ion milling system). For the precise stopping of the etching at the Pt layer, in situ SIMS was used during the etching process. The etching was stopped when the Co signal was reduced by 50% and the Pt signal was increased to 50% of its final value (full width at half-maximum (fwhm)). Finally, a lift-off technique (maskless UV lithography in a MLA150 with the photoresist, ARP3540T) was used to create electrical contact pads (30Ti-750Au via ion beam deposition, scia coat 200).

### Discussion on DW Spacing Compression and Decompression

Here, we further discuss the DW spacing compression and decompression. For simplicity, we assume that the current density, *J*, changes at *x* = *x*_0_ (*J*-boundary) so that the DW speed changes at *x* > *x*_0_, as shown in Figure S8. Also, we assume that the DW speed is uniform for a fixed *J* (i.e., no defects). For a bit, there exist two DWs with a spacing (*l*), which is defined by the difference in positions of the two DWs, i.e., *l* = |*x*_1_ – *x*_2_|. Under uniform *J*, both DWs move at the same speed (*v*_1_ = *v*_2_), i.e., *l* does not change upon the CIDWM. However, as the first DW shifts across the *J*-boundary, the speed difference between DWs reduces the DW spacing if *v*_1_^′^ < *v*_2_ (compression condition) or enlarges the DW spacing if *v*_1_^′^ > *v*_2_ (decompression condition). Eventually, as the second DW shifts across the *J*-boundary, the final DW spacing, *l*′, becomes *v*_1_^′^*t*_1_ where *t*_1_ is the elapsed time count until DW2 arrives at the boundary following DW1. The final DW spacing can be simply expressed by
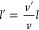
where *l*′, *v*′, *v*, and *l* denote the final DW spacing, DW speed after the boundary, DW speed before the boundary, and initial DW spacing.

## Supporting Information Available

Further device details (magnetization, fabrication, *v* vs *J*, characteristics of multisection devices, additional explanation on compression and decompression), and discussion on *v* vs *J* (PDF)
Bit compression in ferromagnetic nanowire (MP4)
Bit compression in synthetic antiferromagnetic nanowire (MP4)
4-Bit compression in synthetic antiferromagnetic nanowire (MP4)
