# Supplementary material for: Dynamic Manipulation of Chiral Domain Wall Spacing for Advanced Spintronic Memory and Logic Devices
Source: ACS Nano. 2024 May 17;18(22):14507–13. doi: 10.1021/acsnano.4c02024 (PMC11155243; doi:10.1021/acsnano.4c02024)
Supplement: Supplementary file 1 — nn4c02024_si_001.pdf [file nn4c02024_si_001.pdf]

Supporting Information

Dynamic manipulation of chiral domain wall spacing for advanced spintronic memory and logic devices

Jae-Chun Jeon[1,3,*], Andrea Migliorini[1,3], Lukas Fischer[1,2], Jiho Yoon[1], and Stuart S. P. Parkin[1,*]

[1]Max Planck Institute for Microstructure Physics, Halle (Saale), D-06120, Germany

[2]Martin Luther University Halle-Wittenberg, Halle (Saale), 06108, Germany

[3]These authors contributed equally to this work

*Corresponding authors: jae-chun.jeon@mpi-halle.mpg.de, stuart.parkin@mpi-halle.mpg.de

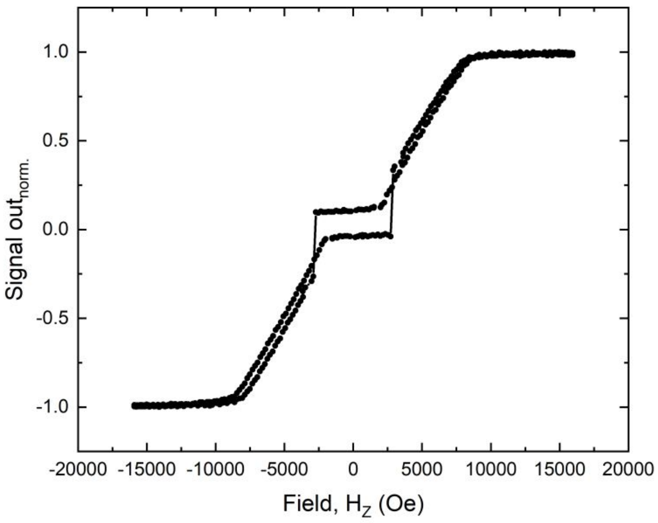

Figure S1. Polar magneto-optical Kerr spectroscopy of a typical synthetic antiferromagnetic film.

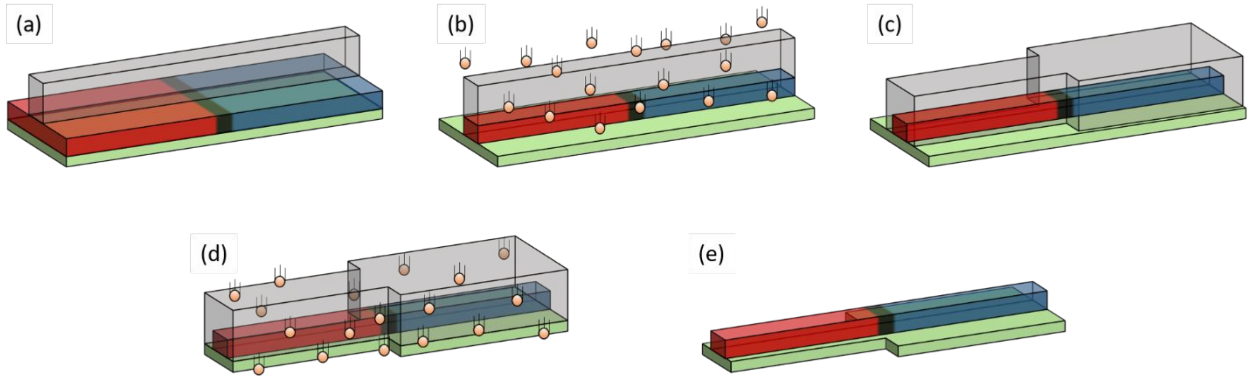

Figure S2. Illustrations of device fabrication process: (a) Photo/e-beam resist coating and lithography patterning. At this stage, straight racetrack is defined. (b) Ar-ion beam etching process. The etching was precisely controlled via in-situ end point detector to stop at Pt layer. (c) Resist coating for the 2nd layer to define sections. (d) 2nd Ar-ion beam etching. At this stage, the entire layer of the film is etched away. (e) Final scheme of the device.

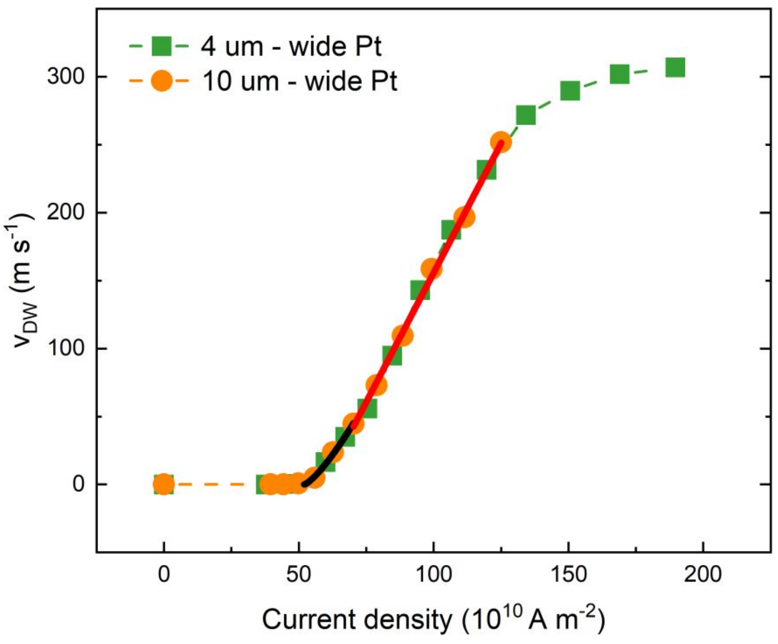

Figure S3. Domain wall speed vs. current density in each section of the 2-sectioned (4 μm- and 10 μm-wide spin Hall layer with 2 μm-wide SAF layer) device. Black and red lines are the fitting curves in depinning and propagation regions.

Discussion on v_{CIDWM} vs. J

Current induced domain wall motion is often simulated by by solving the Landau-Lifshitz-Gilbert (LLG) equations assuming that the DW shape does not change during its motion. In this 1-D model, the analytical expression of the DW velocity can be derived under the weak perturbation condition, i.e., $sin\psi \sim \psi$ & $\dot{\psi} = 0$. The expressions for the DW velocity, $v = \dot{q}$, in the ferromagnet (FM) and synthetic antiferromagnet (SAF) cases are given by the following equations[6, 10];

$$\dot{q}(FM) = \frac{\beta}{\alpha}u + \frac{\pi\Delta\gamma}{2\alpha}H_{SHE}sin\psi$$

where $H_{SHE} = \frac{\hbar}{2eM_S t}J_s = \frac{\hbar\theta_{SH}J}{2eM_S t}$, $u = \frac{\mu_B PJ}{eM_S}$, α is the Gilbert damping constant, β is the spin transfer torque constant, Δ is the domain wall width, γ is the gyroscopic ratio, P is the spin polarization of the current, M_S is the saturation magnetization, and θ_{SH} is the spin Hall angle, respectively.

$$\dot{q}(SAF) = \frac{\alpha_L \alpha_U}{\alpha_L M_L(1+\alpha_L^2)+\alpha_U M_U(1+\alpha_U^2)}\left[-M_L\left(\frac{1}{\alpha_L}+\beta_L\right)u_L - M_U\left(\frac{1}{\alpha_U}+\beta_U\right)u_U\right] \pm \frac{\gamma \Delta M_L}{\alpha_L}\left\{\frac{H_L^k}{2} sin2\psi_L - \frac{\pi}{2} H_L^{lg} sin\psi_L - \frac{2J^{ex}}{M_L}\sin{(\psi_L - \psi_U)} + \frac{\alpha_L \pi H_L^{SHE}}{2} cos\psi_L\right\} \pm \frac{\gamma \Delta M_U}{\alpha_U}\left\{\frac{H_U^k}{2} sin2\psi_U - \frac{\pi}{2} H_U^{lg} sin\psi_U - \frac{2J^{ex}}{M_U}\sin{(\psi_U - \psi_L)} + \frac{\alpha_U \pi H_U^{SHE}}{2} cos\psi_U\right\}$$

where U and L denote the upper and lower layers within a SAF structure, $H_i^k = 2K_i^u t_i/M_i$ ($i = L$ and U for lower and upper layers), and $H_i^{lg} = H_x + H_i^{DMI}$, respectively.

In these simplified analytical models the threshold current density, J_{th}, is zero. However, phenomenologically, there always exist creep, pinning, de-pinning, propagation, and Joule heating for the current induced domain wall motion (CIDWM) in racetrack devices, which are not included in the model. For creep and de-pinning, the non-linear DWM can be expressed by [Ref S1]

$$\dot{q}(creep) \sim \exp\left[-\frac{U_C}{T}\left(\frac{J}{J_C}\right)^{-\mu}\right]$$

where $\mu = \frac{D-2+2\xi_{eq}}{2-\xi_{eq}}$, D is the dimensional constant, ξ_{eq} is the roughening exponent, U_C is the energy scale of a Larkin domain[Ref. S1]. Note that creeping of a DW occurs over much longer time-scales than nano-seconds. Therefore, for the CIDWM experiments with ns-pulses, we often consider $\dot{q} = 0$ in this range, i.e., $0 < J < 50$ MA/cm^2 in Fig. S3.

$$\dot{q}(depinning) \sim (J - J_C)^{\eta} \text{ for } J > J_C$$

In the depinning region, i.e., 50 MA/cm^2 $< J <$ 70 MA/cm^2 in Fig. S3, we obtain J_C = 52 MA/cm^2 and $\eta = 1.3$, respectively.

In the linear motion region (propagation region), the CIDWM can be expressed by

$$\dot{q}(linear) \sim \zeta J$$

For 70 MA/cm^2 $< J <$ 125 MA/cm^2, ζ is found to be 3.8 for our device (Fig. S3).

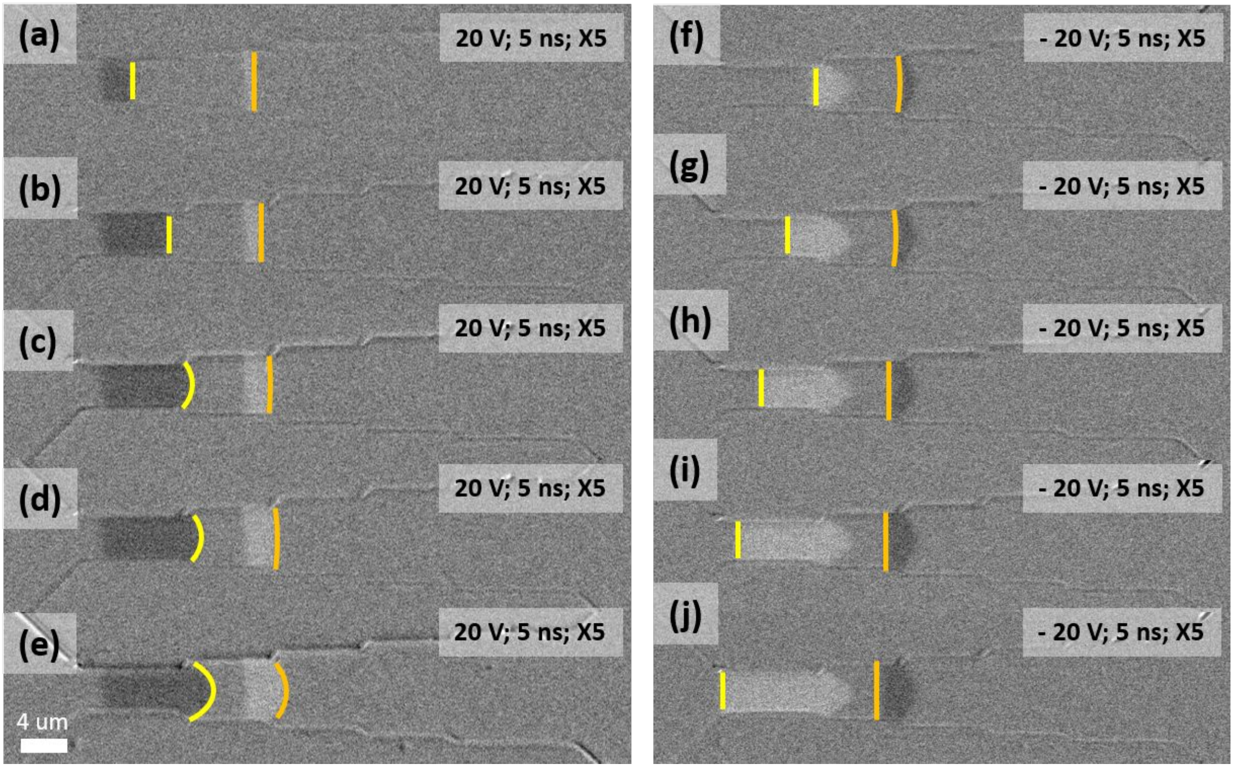

Figure S4. Differential Kerr microscope images for multi-section device. (a) – (e) Forward propagation of two DWs. The DWs strongly pin at each steps, which blocks the continuous propagation of DWs along the magnetic wire. (f) – (j) Backward propagation of two DWs. Note that due to the pinning of the DW at the steps at the edges, the DW becomes curved when current pulses are applied. Such a curvature on the DW increases its energy due to lengthening of the DW which determines the extent of the curving.[Refs S2, S3] When the injection of the current pulses was stopped, a slow return of the DW to its previous uncurved shape was found.

Attempt - 1		
Pt-wing width (ratio)	Expected DW spacing (μm)	Measured DW spacing (μm)
4 μm (1:1)	5.9	5.9 ± 0.5
8 μm (1:2)	4.7	3.8 ± 0.5
12 μm (1:3)	3.5	2.7 ± 0.5
16 μm (1:4)	2.4	2 ± 0.5
20 μm (1:5)	1.2	1.2 ± 0.5
**Attempt - 2**		
Pt-wing width (ratio)	Expected DW spacing (μm)	Measured DW spacing (μm)
4 μm (1:1)	8.1	8.1 ± 0.5
8 μm (1:2)	6.5	6.4 ± 0.5
12 μm (1:3)	4.9	4.2 ± 0.5
16 μm (1:4)	3.2	2.1 ± 0.5
20 μm (1:5)	1.7	1 ± 0.5
**Attempt - 3**		
Pt-wing width (ratio)	Expected DW spacing (μm)	Measured DW spacing (μm)
4 μm (1:1)	8.1	8.6 ± 0.5
8 μm (1:2)	6.5	6.9 ± 0.5
12 μm (1:3)	4.9	3.3 ± 0.5
16 μm (1:4)	3.2	1.4 ± 0.5
20 μm (1:5)	1.7	1 ± 0.5

Table S1. Comparison between expected DW spacing and measured DW spacing in the multi-step device. The calculation is made by the assumption that the v_{CIDWM} is linearly proportional to the current density. In 3 attempts, the mean value of the difference is ~ 18 %.

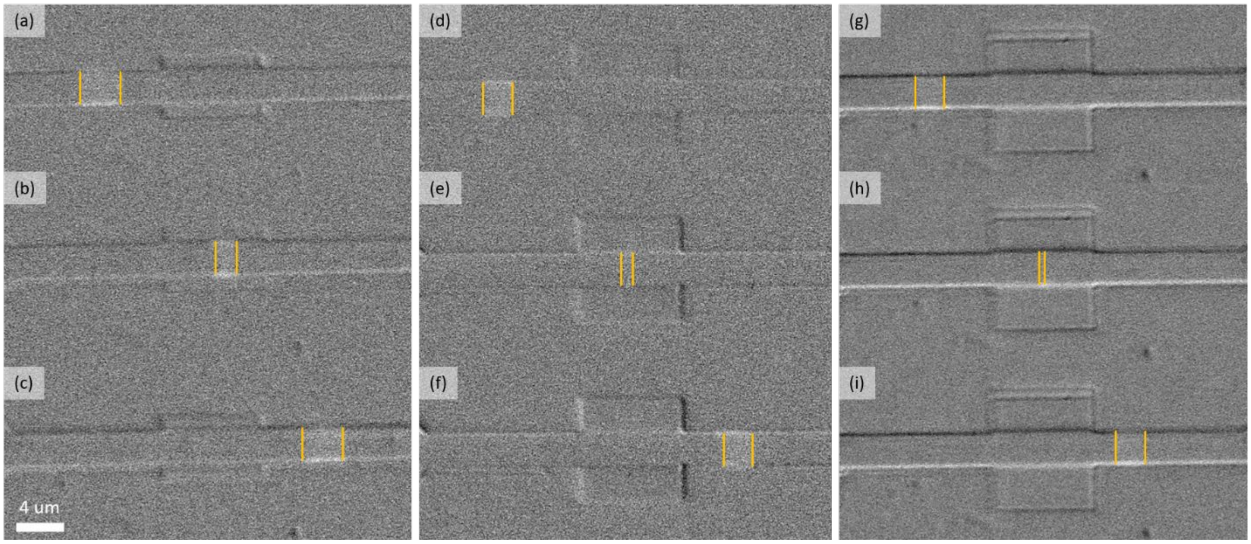

Figure S5. Decompress-compress-decompress type devices with different racetrack vs. Pt wing ratio. (a) – (c) 1 : 2 (3 μm : 6 μm); (d) – (f) 1 : 3.3 (3 μm : 10 μm); (g) – (i) 1 : 4 (3 μm : 12 μm). (a), (d) and (g) show the initial two domain walls. (b), (e) and (h) show the Kerr images when two DWs are positioned in the middle of the compress region. (c), (f) and (i) show the DW spacing when two DWs enter into the decompress region. For the experiments, 14 V; 10 ns; 2 consecutive pulses were used to shift the DWs. Orange lines mark the position of the DWs.

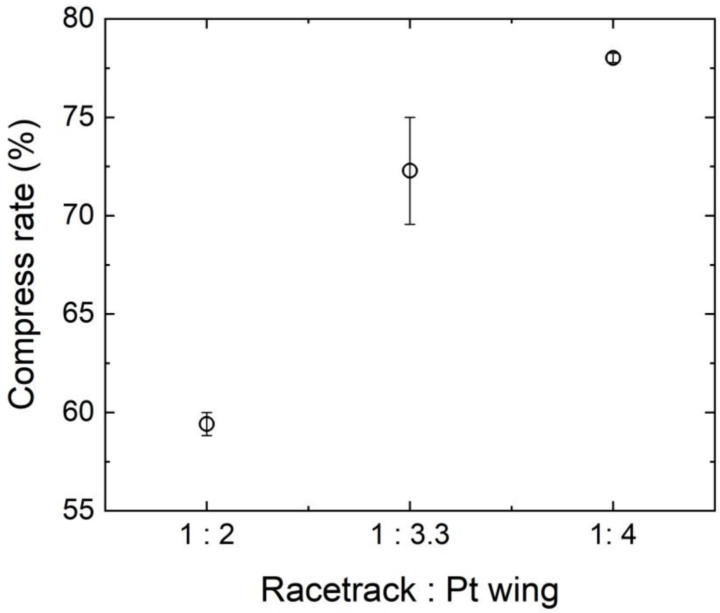

Figure S6. Compress rate vs. racetrack : Pt wing ratio. The data is extracted from Fig. S5.

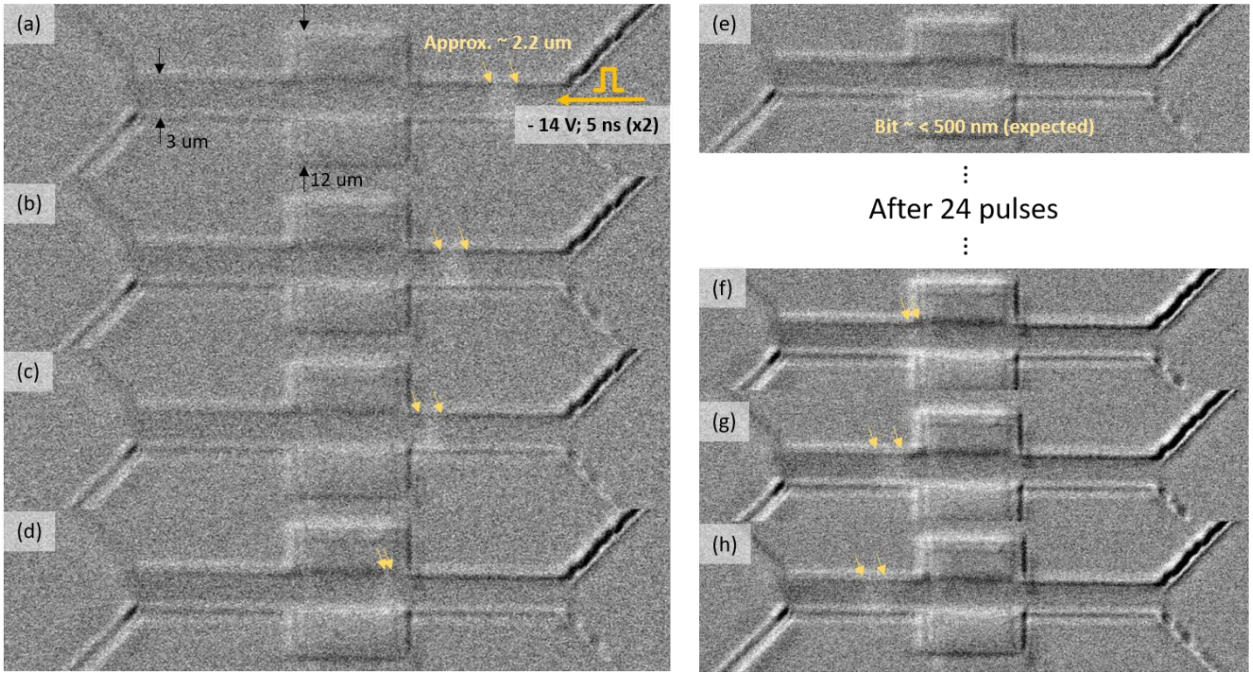

Figure S7. Passive control of bit compress beyond the resolution of the Kerr microscope. Two domain walls move from right to left by - 14 V; 5 ns; 2 pulses. (a) - (c) DW motion in the decompress region. (d) – (f) DW spacing reduction in the compress region. Note that the DWs are invisible when the bit is placed within the compressive region (expected bit size ~ 480 nm – considering the compress ratio of 78 %). As the DWs escape the compressive region and enters into decompress region, it recovers the original DW spacing (~ 2.2 μm).

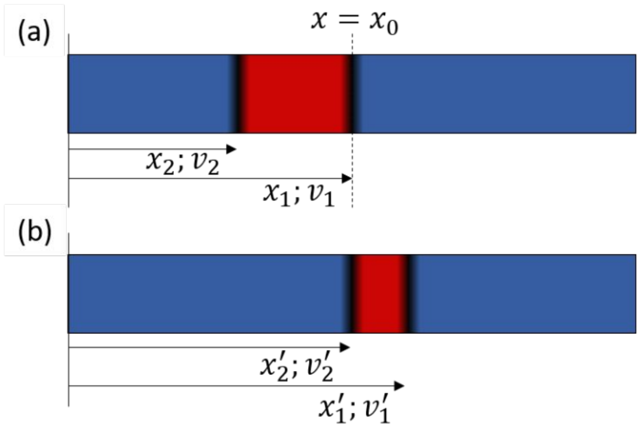

Figure S8. Illustrative explanation of domain bit compression. x_1,v_1, x_2, and v_2 denote the positions and local speeds of DW1 and DW2 at $t = t_0$ (x_1, $x_2 < x_0$). x_1',v_1',x_2', and v_2' denote the positions and local speeds of DW1 and DW2 at $t = t_1$ (x_1', $x_2' > x_0$). In this example, we assume $v_i = 2v_i'$ at $x < x_0$. (a) The moment when DW1 arrives at the *J*-boundary ($x = x_0$). From this moment, DW1 moves twice slower than DW2 which continuously compresses the spacing between DWs. (b) When DW2 enters into the *J*-boundary, DW1 and DW2 moves at the same speed and the compression stops. Note that the compression rate is proportional to the DW speed difference across the *J*-boundary.

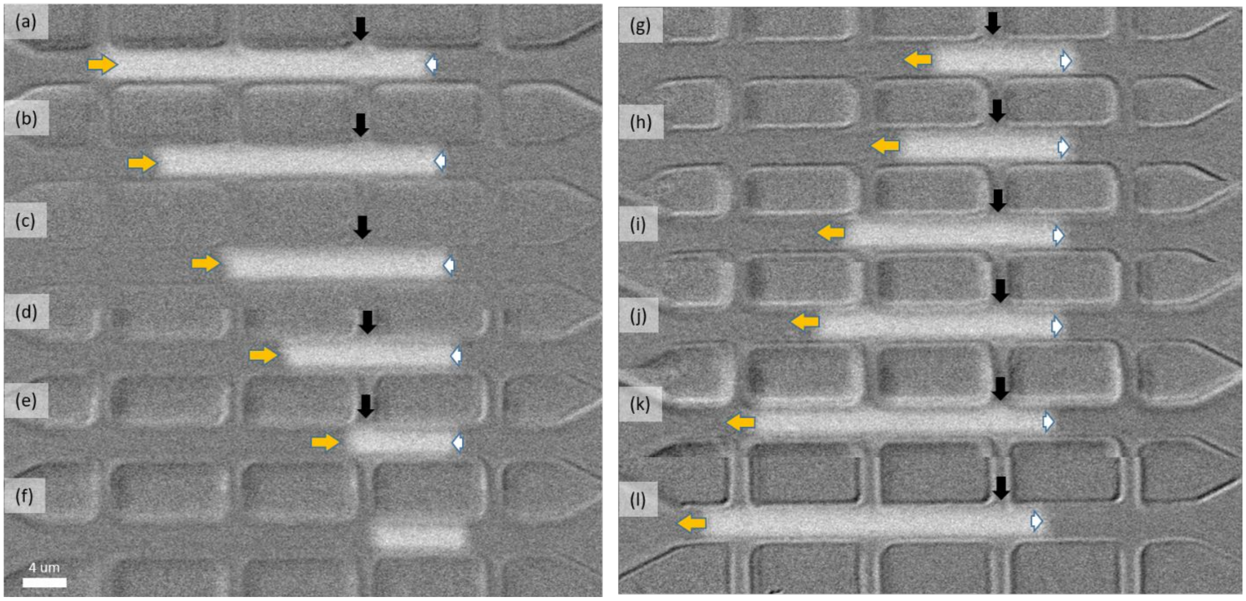

Figure S9. Control of the DW spacing via local bias. (a) – (f) Compression of DW spacing with a reverse bias (V_{bias} = - 0.5 V) – positive current pulse (18 V; 10 ns). 29.5 μm of initial spacing between two DWs is compressed to 9.8 μm via the operation. (g) - (l) Decompression of the DW spacing with forward bias (V_{bias} = 0.5 V) – negative current pulse (- 18 V; 10 ns). Note that the corresponding current density (5 MA cm^{-2}) of the bias level is lower than the threshold current density for current induced domain wall motion (J_{th} = 40 MA cm^{-2}). Black, yellow, and white arrows indicate the bias point, direction of DWM, direction of bias, respectively.

Supplementary references

Ref S1) Chauve, P., Giamarchi, T., and Le Doussal, P., *Creep and depinning in disordered media.* Phys. Rev. B 2000 62, 6241.

Ref S2) Zhang, Y., *et al*. *Domain-Wall motion driven by Laplace pressure in Co-Fe-B / MgO nanodots with perpendicular anisotropy*. Phys. Rev. Appl. 2018 9, 064027.

Ref S3) Zhang, X., et al. *Direct observation of domain-wall surface tension by deflating or inflating a magnetic bubble*. Phys. Rev. Appl. 2018 9, 024032.

Captions for supplementary videos

VS1_FM_2DW.mp4 The motion capture of the results from micromagnetic simulation for a bit compression via local current density control in a ferromagnetic nanowire (Fig. 5b).

VS2_SAF_2DW.mp4 The motion capture of the results from micromagnetic simulation for a bit compression via local current density control in a synthetic antiferromagnetic nanowire (Fig. 5a).

VS3_8DW_SAF.mp4 The motion capture of the results from micromagnetic simulation for 4-bit compression via local current density control in a synthetic antiferromagnetic nanowire (Fig. 5c).
